# Development and validation of a risk prediction score for severe acute pancreatitis

**DOI:** 10.1186/s12967-019-1903-6

**Published:** 2019-05-08

**Authors:** Wandong Hong, Keith D. Lillemoe, Shuang Pan, Vincent Zimmer, Evangelos Kontopantelis, Simon Stock, Maddalena Zippi, Chao Wang, Mengtao Zhou

**Affiliations:** 1 Department of Gastroenterology and Hepatology, The First Affiliated Hospital of Wenzhou Medical University, Nanbaixiang, Ouhai District, Wenzhou, 325000 Zhejiang People’s Republic of China; 2 Department of Surgery, Massachusetts General Hospital Harvard Medical School, Boston, MA 02114 United States; 3 Department of Gastroenterology and Hepatology, The First Affiliated Hospital of Wenzhou Medical University, Wenzhou, Zhejiang People’s Republic of China; 4 Department of Medicine II, Saarland University Medical Center, Saarland University, 66424 Homburg, Germany; 5 Department of Medicine, Marienhausklinik St. Josef Kohlhof, 66539 Neunkirchen, Germany; 6 Division of Informatics, Imaging and Data Science, Faculty of Biology, Medicine and Health, University of Manchester, Manchester, M13 9GB UK; 7 NIHR School for Primary Care Research, Centre for Primary Care and Health Services Research, University of Manchester, Manchester, UK; 8 Department of Surgery, World Mate Emergency Hospital, Battambang, Cambodia; 9 Unit of Gastroenterology and Digestive Endoscopy, Sandro Pertini Hospital, Rome, Italy; 10 Department of Gastroenterology, The First Affiliated Hospital of Soochow University, Jiangsu, People’s Republic of China; 11 Department of Surgery, Key Laboratory of Diagnosis and Treatment of Severe Hepato-Pancreatic Diseases of Zhejiang Province, The First Affiliated Hospital of Wenzhou Medical University, Wenzhou, Zhejiang People’s Republic of China

**Keywords:** Prediction, Acute pancreatitis, Severity, Risk factor, Score

## Abstract

**Introduction:**

The available prognostic scoring systems for severe acute pancreatitis (SAP) have limitations that restrict their clinical value. The aim of this study was to develop a simple model (score) that could rapidly identify those at risk for SAP.

**Methods:**

We derived a risk model using a retrospective cohort of 700 patients by logistic regression and bootstrapping methods. The discriminative power of the risk model was assessed by calculating the area under the receiver operating characteristic curves (AUC). The classification and regression tree (CART) analysis was used to create risk categories. The model was internally validated by a tenfold cross-validation and externally validated in a separate prospective cohort of 194 patients.

**Results:**

The incidence of SAP was 9.7% in the derivation cohort and 9.3% in the validation cohort. A prognostic score (We denoted it as the SABP score), ranging from 0 to 10, consisting of systemic inflammatory response syndrome, serum albumin, blood urea nitrogen and pleural effusion, was developed by logistic regression and bootstrapping analysis. Patients could be divided into three risk categories according to total SABP score based on CART analysis. The mean probability of developing SAP was 1.9%, 12.8% and 41.6% in patients with low (0–3), moderate (4–6) and high (7–10) SABP score, respectively. The AUCs of prognostic score in tenfold cross-validation was 0.873 and 0.872 in the external validation.

**Conclusion:**

Our risk prediction score may assist physicians in predicting the development of SAP.

**Electronic supplementary material:**

The online version of this article (10.1186/s12967-019-1903-6) contains supplementary material, which is available to authorized users.

## Introduction

Though most patients with acute pancreatitis (AP) suffer from a mild and self-limiting form with a benign clinical course [1, 2]. Approximately 10–20% of all cases present with severe acute pancreatitis (SAP), which is associated with a significant risk of mortality [3, 4].

Early identification of high-risk patients on admission may help physicians to select those patients who would benefit the most from close surveillance, or aggressive intervention [5]. Firstly, early identification of patients who have a high probability of developing SAP in the emergency room may benefit from close surveillance, aggressive critical care and early treatment [5, 6]. Early aggressive intravenous hydration is most beneficial in the first 12–24 h and may have little benefit beyond this time [2, 7]. Early fluid resuscitation may improve microcirculation of the pancreas and provide hemodynamic support, which results in reduced incidence of morbidity and mortality among patients with acute pancreatitis [8, 9]. Early endoscopic retrograde cholangiopancreatography (ERCP) is associated with fewer complications in predicted severe acute biliary pancreatitis [10]. Secondly, it was reported that patients in high-volume centers had a shorter length of stay, lower hospital charges, and lower mortality rates than do those in low-volume centers [11]. As a result, clinicians need to identify those patients who do not respond to early resuscitation or display SAP for possible transfer to specialist care or a pancreatitis centre if available [2, 6]. Lastly, the ability to identify patients at risk of SAP early in the disease course also helps in designing mechanistic studies or clinical trials for targeted intervention [3].

Many clinical scoring systems have been developed, such as the Bedside index of severity in acute pancreatitis (BISAP) [12],chronic health evaluation (APACHE-II) score, and modified Glasgow score, Japanese severity score (JSS) [13], and the Harmless acute pancreatitis score (HAPS) [14]. However, these existing scoring systems were primarily derived for prediction of severe disease based on the Atlanta criteria or mortality but not for SAP defined by recent revised international guidelines on acute pancreatitis [3, 6]. Although individual predictors, such as admission hematocrit (≥ 44%) or blood urea nitrogen (BUN) at 24 h, are easy to use in practice, they lack high sensitivity or specificity [15].

Therefore, the aim of this work was to develop and validate a simple risk score for the early prediction of SAP.

## Patients and methods

### Inclusion and exclusion criteria

Patients with acute pancreatitis admitted to the First Affiliated Hospital of Wenzhou Medical University (Wenzhou City, Zhejiang Province, China) within 72 h of symptom onset from January 2012 to December 2015 were retrospectively included in the derivation cohort [16]. Thereafter, patients with acute pancreatitis from January 2016 to December 2016 in the First Affiliated Hospital of Soochow University (Suzhou City, Jiangsu Province, China) were prospectively included in the validation cohort. Acute pancreatitis was defined as previously described [1, 6]. According to the revised Atlanta classification, SAP is characterized by single or multiple organ failure (respiratory, cardiovascular, renal) that persists for > 48 h [2, 6].

Exclusion criteria were [16]; patients that had developed organ failure before data collection, previous pancreatic surgery, recurrent or not first-time pancreatitis, pancreatitis due to endoscopic retrograde cholangiopancreatography (ERCP) or trauma, chronic pancreatitis, pancreatic cancer, pleural effusions both preceding the development of AP and as the result of concomitant diseases (e.g., pneumonia, chronic heart failure), chronic renal disease, patients with albumin infusion before data collection in our hospital, hypoalbuminemia due to malnutrition, albuminuria, hepatitis, liver cirrhosis.

### Data collection

Age, gender, body mass index (BMI), time from symptom onset to admission and biochemical parameters were recorded within 12 h of hospitalization, except for serum albumin levels which were assayed within the first 24 h [16]. All patients underwent abdominal computed tomography (CT) scan within 6 h of admission and the presence of a pleural effusion was recorded. Data for every variable of systemic inflammatory response syndrome (SIRS), BISAP, APACHE II, HAPS, Glasgow and JSS scores were collected if available and were calculated as described by Wu et al. [12] and Mounzer et al. [3].

### Statistical analysis

Categorical variables were described using frequencies and proportions and compared using χ2 tests. Continuous values were expressed using mean ± standard deviation (SD), or median and interquartile range (IQR) and compared using Student’s *t* test or the nonparametric Mann–Whitney test. Linear trend of categorical and continuous variables was tested using a Royston extension of the Cochran–Armitage test [17] and a non-parametric Wilcoxon rank sum test [18], respectively.

For easier application to a risk score model, when performing multivariate logistic regression analysis, most continuous variables were converted to categories based on published data as follows: advanced age (≥ 60 years) [12], body mass index (BMI) (≥ 30) [19], SIRS (yes vs. no) [12], hematocrit (≥ 44%) [15], platelet count (≤ 100,000/mm^3^) [20], prothrombin time (PT ≥ 18 s) [21], total bilirubin (≥ 2 mg/dL), alanine aminotransferase (ALT > 50 U/L), aspartate aminotransferase (AST > 45 U/L) [22], glucose (≥ 150 mg/dL) [19], blood urea nitrogen (BUN) (> 25 mg/dL) [12] and pleural effusion (yes vs. no) [5].

Candidate predictors with P < 0.20 in univariate analyses were included a multivariate logistic regression. In addition, a backward stepwise bootstrap regression model, in which 1000 random samples patients were generated with replacement, was also performed to investigate the relative importance of each variable included in our model [23]. Frequencies of occurrence of each covariate in the final model were noted; if predictors occurred in 90% or more of the bootstrap models, they were retained in the final multivariate model [24]. Beta regression coefficients and odds ratios (OR) were calculated with 95% confidence intervals (CI). The multivariate regression coefficients of the predictive factors were used to assign integer points for the prediction score [25, 26]. Individual risk estimates were based on the sum of weighted scores for each variable.

The discriminative power of the prediction score was assessed by calculating the area under the receiver operating characteristic (ROC) curves (AUC) [27]. All variables were used as continuous variables when calculating AUC. A predictor with an AUC above 0.7 was considered to be useful, while an AUC between 0.8 and 0.9 indicated good diagnostic accuracy [28].

The model was internally validated using tenfold cross-validation [29, 30]. When performing tenfold cross-validation, we first randomly divided all data into ten equal-sized subsamples. The aim is to use nine subsamples for training and the remaining one for testing, over all possible permutations. Through the cross-validation process, the analysis is then repeated ten times (folds), with each of the ten subsamples used exactly once as the validation data [30]. The AUC is calculated for each of the 10 analyses, using only the respective test data, and these 10 AUC statistics are then further aggregated into means, standard deviation (through which 95% confidence intervals are calculated), medians, etc. [29]. The classification and regression tree (CART) analysis was used to create risk categories according to total prediction score [5]. When performing CART analysis, impurity function was used for splitting and cut-off points for continuous variables which were generated automatically based on statistical cost assumptions [5]. Calibration of the risk score reflecting the link between predicted and observed risk, was evaluated by the Hosmer–Lemeshow goodness of fit test [31].

A P value < 0.05 was considered statistically significant for all analyses. Data were analyzed using the STATA version 12 and R 3.5.1 statistical software.

## Results

### Characteristics of the investigated population

Distributions for demographic and clinical features between the two study populations are depicted in Table 1. There were 700 and 194 patients enrolled in the derivation cohort and validation cohort respectively. Biliary cause was the most common etiology in 42.7% of patients in the derivation cohort and 38.7% of patients in the validation cohort. The incidence of SAP was 9.7% (68/700) and 9.3% (18/194) in the derivation cohort and validation cohort respectively.

**Table 1:** Baseline characteristics and risk factors in derivation and validation cohorts

Variable	Derivation cohort (n = 700)	Validation cohort (n = 194)
Age, years (IQR)	48 (37–63)	49 (38–61)
Male sex, N (%)	435 (62.1)	127 (65.5)
Duration of symptoms, days	1.83 ± 0.79	1.67 ± 0.78
SIRS, N (%)	272 (38.9)	82 (42.3)
Etiology		
Biliary, N (%)	299 (42.7)	74 (38.1)
Alcohol, N (%)	96 (13.7)	12 (6.2)
Hypertriglyceridemia, N (%)	37 (5.3)	31 (16.0)
Idiopathic, N (%)	246 (35.1)	73 (37.6)
Other, N (%)	22 (3.1)	4 (2)
Laboratory findings		
Hematocrit	0.42 (0.38–0.46)	0.43 (0.39–0.46)
Platelets (10^9^/L)	197 (158–233)	206 (169–248)
Prothrombin time, s (IQR)	13.8 (13.1–14.6)	13.7 (13.2–14.5)
Albumin, g/L (IQR)	36.2 (32.9–39.7)	36.6 (32.7–39.7)
Bilirubin, mg/dL (IQR)	1.16 (0.79–1.81)	1.11 (0.76–1.75)
ALT, U/L (IQR)	40 (19–107)	33 (18–91)
AST, U/L (IQR)	36 (21–85)	33 (23–73)
Glucose, mg/dL (IQR)	144 (117–193)	139 (112–191)
BUN, mg/dL (IQR)	13.4 (10.4–17.4)	14.0 (10.6–17.9)
Pleural effusion, N (%)	135 (19.4)	83 (42.8)
Clinical outcomes		
Patients with SAP, N (%)	68 (9.7)	18 (9.3)
Number of POF, N (%)		
One organ failure	38/68 (55.9)	7/18 (38.9)
Two organ failure	19/68 (27.9)	6/18 (33.3)
Three organ failure	11/68 (16.2)	5/18 (27.8)
Type of POF		
Respiratory failure, N (%)	56/68 (82.4)	16/18 (88.9)
Renal failure, N (%)	24/68 (35.3)	10/18 (55.6)
Cardiovascular failure, N (%)	29/68 (42.7)	8/18 (44.4)
Length of hospital stay, days (IQR)	10 (7–9)	11 (7–17)
Hospital mortality, N (%)	11 (1.6)	2 (1.0)

Data were mean ± standard deviation, or numbers and percentages, or median (25th–75th percentile), as appropriate

N, number; IQR, interquartile range; BMI, body mass index; SIRS, systemic inflammatory response syndrome; ALT, alanine aminotransferase; AST, aspartate aminotransferase; BUN, blood urea nitrogen; SAP, severe acute pancreatitis; POF, persistent organ failure

### Univariate and multivariate analysis in the derivation cohort

Sixteen variables considered relevant to the presence of SAP were tested using univariate analysis (Additional file 1: Table S1). Age, BMI, alcohol etiology, SIRS, hematocrit, platelet count, prothrombin time, albumin, AST, glucose, BUN and pleural effusion were identified as candidate predictors (P < 0.20) of SAP in univariate analysis. When using these potential predictors (except albumin) as categories, all remained independently associated with SAP in multivariate logistic regression analysis: SIRS (odds ratios (OR) 2.98; 95% confidence interval (CI) 1.47–6.04; P = 0.003), hematocrit (OR 2.24; 95% CI 1.13–4.46; P = 0.021), albumin (OR 0.51; 95% CI 0.36–0.73; P < 0.001), AST (OR 2.18; 95% CI 1.13–4.19; P = 0.02), serum glucose (OR 2.27; 95% CI 1.12–4.61; P = 0.023), BUN (OR 4.58; 95% CI 2.16–9.70; P < 0.001) and pleural effusion (OR 4.68; 95% CI 2.42–9.05; P < 0.001).

### Bootstrap analysis of potential predictors and development of prediction score in the derivation cohort

The bootstrap analysis revealed that, out of twelve potential predictors, SIRS, albumin, BUN and pleural effusion were reproducibly selected in more than 90%. Therefore, these four variables were kept in the final model for the development of the prediction score. The final logistic regression function was: log (odds of SAP) = 0.55 + 1.02 (SIRS)–0.63 (albumin) + 1.76 (BUN) + 1.66 (pleural effusion). The logistic regression coefficients and 95% CI, as well as the allocation of scoring points for each predictive factor based on the regression coefficients, are given in Table 2. We denoted it as the SABP (SIRS, albumin, BUN and pleural effusion) score. The total prediction score ranges between 0 and 10 with a high score indicating high risk of developing SAP.

**Table 2:** Point allocation for predictors of severe acute pancreatitis based on regression coefficients

Predictive factor	Regression coefficient	Score assigned
SIRS (yes vs. no)	1.02 (0.39–1.66)	
Yes		2
No		0
Albumin (5 g/L increments)	− 0.63 (− 0.95, − 0.31)	
≥ 35		0
30–34.9		1
< 30		2
BUN (> 25 mg/dL vs. ≤ 25 mg/dL)	1.76 (1.06–2.46)	
> 25 mg/dL		3
≤ 25 mg/dL		0
Pleural effusion	1.66 (1.04–2.27)	
No		0
Yes		3

### Discrimination and internal cross-validation of prediction score in the derivation cohort

**Fig. 1:**
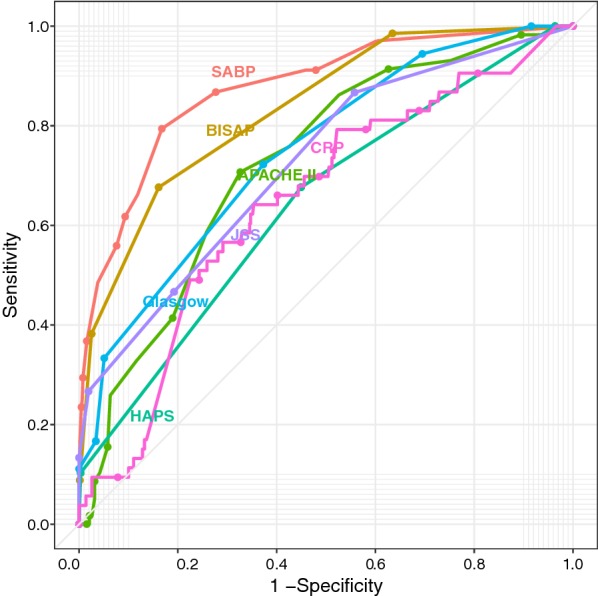
Receiver operating characteristic (ROC) curve for SABP, BISAP, APACHE II (complete data available in 248 patients), HAPS, Glasgow score (complete data available in 77 patients), JSS score (complete data available in 67 patients) and CRP (complete data available in 551 patients) in derivation cohort. AUC, area under the curve of the receiver operating characteristic curve

Based on ROC curve analysis in the derivation cohort (Fig. 1), the SABP score achieved higher AUC than other prediction scoring systems. The AUCs for SABP, BISAP, APACHE II, HAPS, Glasgow score, JSS score and CRP in the prediction of SAP were 0.875 ± 0.023, 0.834 ± 0.024, 0.725 ± 0.037, 0.642 ± 0.032, 0.746 ± 0.063, 0.724 ± 0.073 and 0.646 ± 0.039, respectively.

**Fig. 2:**
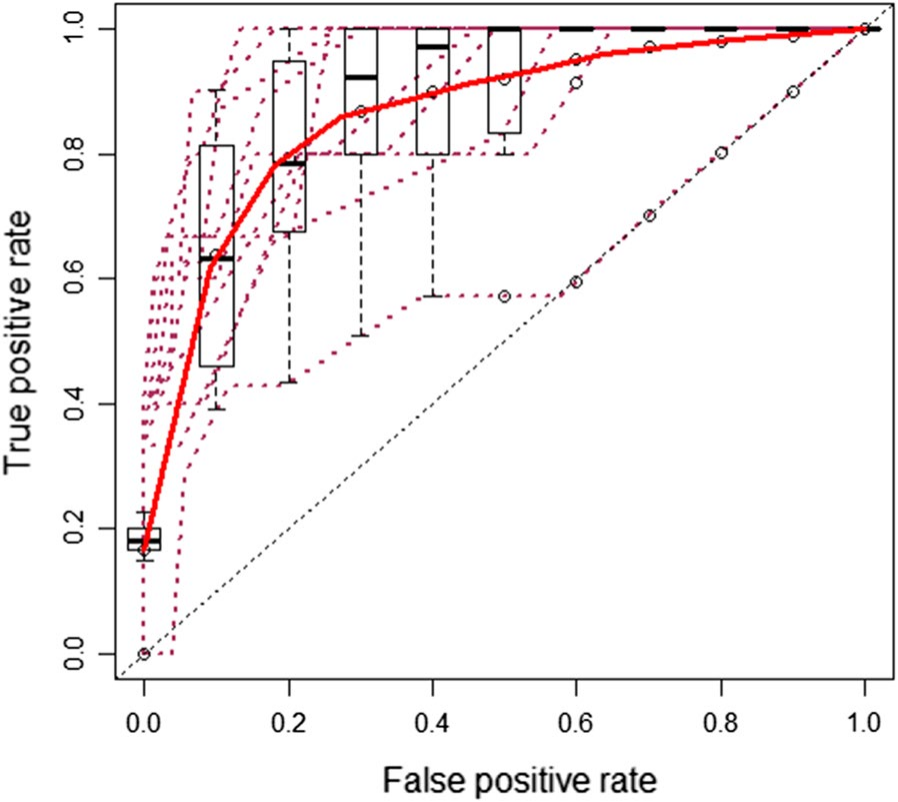
Mean receiver operating characteristic (ROC) curve of SABP score for prediction of severe acute pancreatitis in tenfold cross-validation. Each maroon dash line indicates the receiver operating characteristic (ROC) curve for a validation (testing) data. The red line is the mean ROC curve. The box-plots indicate the variation around the average ROC curve and report the median and the interquartile range

The mean ROC curve of tenfold cross-validation of the SABP score is shown in Fig. 2, which gave an AUC of the 0.873 (95% CI 0.822–0.924) indicating good discrimination for our model. The Hosmer–Lemeshow goodness of fit test of tenfold cross-validation did not reach statistical significance (P = 0.631) indicating a good match of predicted risk over observed risk.

### Application of prediction score in the derivation cohort

**Fig. 3:**
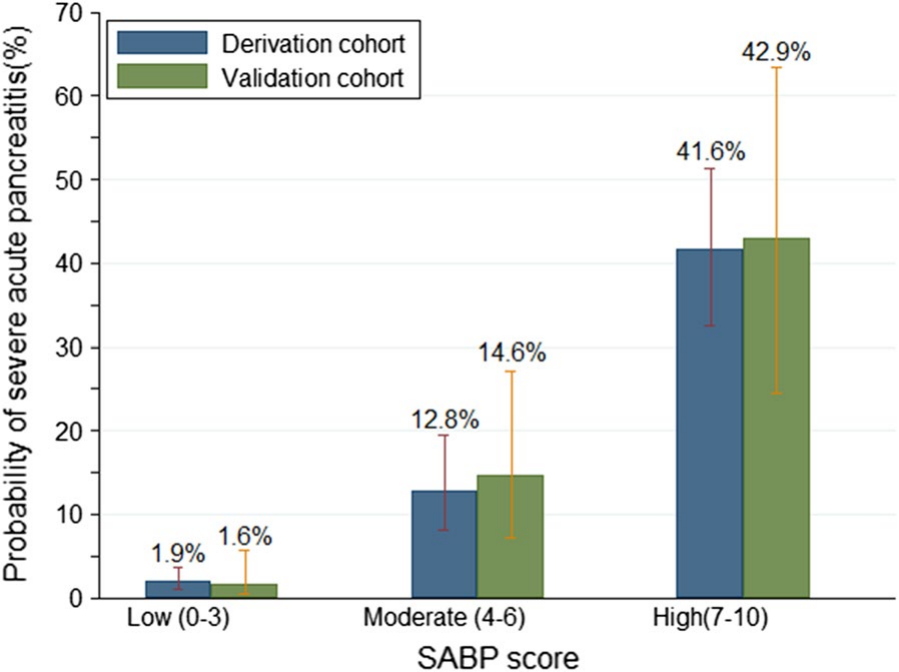
Patients stratified by the SABP score in the derivation cohort and validation cohort according to classification and regression tree (CART) analysis

As shown in Fig. 3, based on CART analysis, patients with acute pancreatitis in the derivation cohort could be divided into three risk categories according to total prediction score: low SABP score (score: 0–3), moderate SABP score (score: 4–6) and high SABP score (score: 7–10). The mean observed probability of developing SAP were 1.9% (9/466), 12.8% (17/133) and 41.6% (42/101) in patients with low, moderate and high SABP score, respectively. This indicated that a higher SABP score was associated with an increased risk of SAP (P_trend_ < 0.001).

**Fig. 4:**
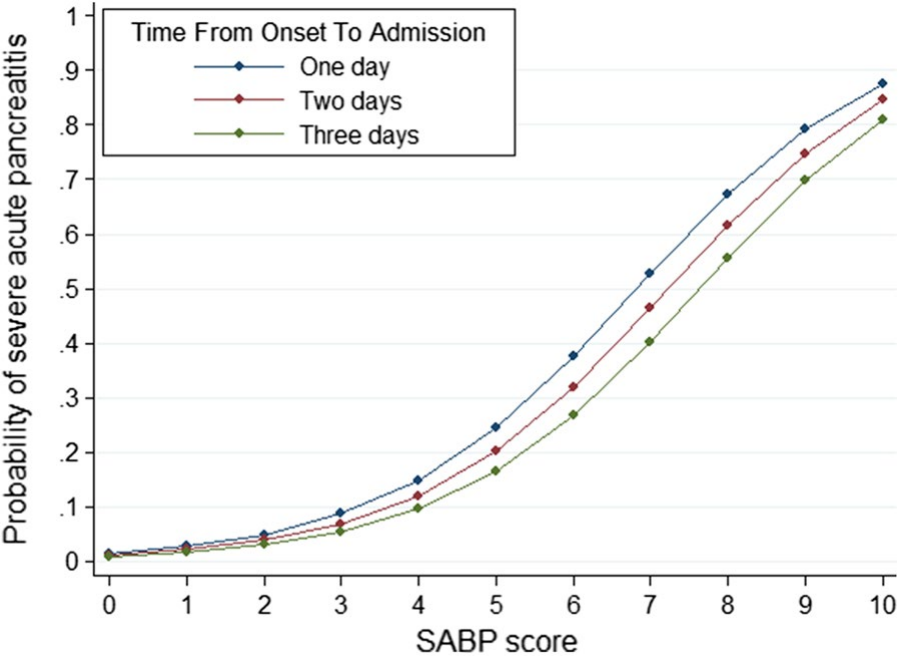
Predicted probability of severe acute pancreatitis in acute pancreatitis over the SABP score from 0 to 10 for different onset-to-admission time

In clinical practice, the time period from onset of pain to hospital admission may play a role in the occurrence and/or severity of SIRS, hypoalbuminemia, pleural effusion and increased BUN [32–34]. Therefore, we computed the predicted probability of SAP over the SABP score from 0 to 10 for different onset-to-admission times (Fig. 4). As an example, using the prevalence of SAP in acute pancreatitis (9.7% in the derivation cohort), a patient with acute pancreatitis admitted to hospital within 1 day after the onset of abdominal pain, with SIRS, a serum albumin level of 33 g/L, BUN level of 26 mg/dL and no pleural effusion would generate a score of 6 points. This translates to a 37.5% probability of developing SAP. However, the probability of developing SAP would decrease to 26.6% for patients with an onset-to-admission time of 3 days with the same score (6 points).

### Performance of prediction score in the validation cohort

**Fig. 5:**
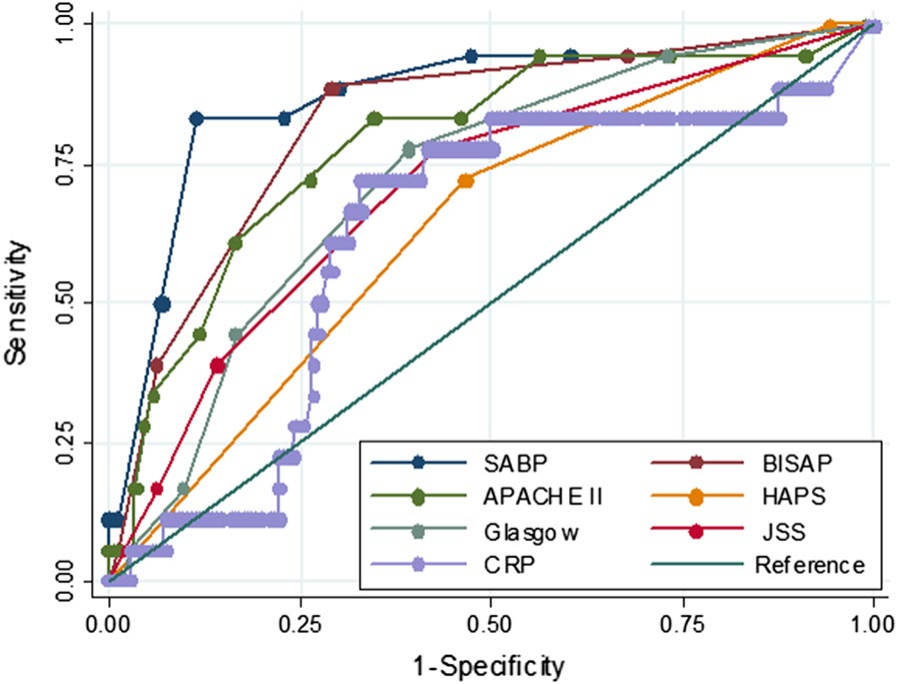
Receiver operating characteristic (ROC) curve for SABP, BISAP, APACHE II, HAPS, Glasgow score, JSS score and CRP in validation. AUC, area under the curve of the receiver operating characteristic curve

Based on ROC curve analysis in the validation cohort (Fig. 5), the SABP score achieved higher AUC than other prediction scoring systems. The AUCs for SABP, BISAP, APACHE II, HAPS, Glasgow score, JSS score and CRP in the prediction of SAP were 0.8725 ± 0.049, 0.8259 ± 0.0497, 0.789 ± 0.059,0.636 ± 0.055, 0.721 ± 0.057,0.703 ± 0.062, 0.623 ± 0.070, respectively.

Using CART analysis, patients with acute pancreatitis in the validation cohort could still be divided into the same three risk categories according to total prediction score) (Fig. 3). The mean observed probability of developing SAP was 1.6% (2/125), 14.6% (7/48) and 42.9% (9/21) in patients with low, moderate and high SABP score, respectively. This indicated that a higher SABP score was associated with an increased risk of SAP (*P*_trend_ < 0.001).

## Discussion

The early extensive systemic release of proinflammatory cytokines, such as interleukin (IL)-1 and IL-6 in patients with acute pancreatitis may give rise to SIRS [35, 36]. Mofidi et al. [35] found that persistent SIRS is associated with multi-organ dysfunction syndrome and death from acute pancreatitis. Singh et al. [36] suggested that patients with a higher number of SIRS criteria on the first day of hospitalization and persistent SIRS had an increased risk of SAP, as defined by persistent organ failure, pancreatic necrosis, need for intensive care unit, and death. Our data suggested that SIRS with an OR of 2.98 (95% CI 1.47–6.04) was independently associated with SAP defined by the up-to-date revised Atlanta criteria.

Hypoalbuminemia may occur in patients with acute pancreatitis due to impaired liver synthesis, increased tissue catabolism and re-distribution from the intravascular to the interstitial space [16]. On the other hand, hypoalbuminemia can lead to the development of pulmonary edema and exacerbation of acute heart failure due to decreased colloid osmotic pressure [37]. Xue et al. [38] suggested that hypoalbuminemia in the early stage was associated with a high incidence of infection and mortality. Our data suggested that an increase of 5 g/L serum albumin level was associated with a statistically significant 49% reduction in the odds of SAP (OR 0.51; 95% CI 0.36–0.73).

A rise in the BUN level at admission in patients with acute pancreatitis may be secondary to pre-renal azotemia due to initial hypovolemia, a state of ongoing negative nitrogen balance related to increased protein catabolism induced by acute pancreatitis, and impairment of renal function [39, 40]. Two large studies have reported that an elevated BUN level at admission is an independent risk factor for mortality in acute pancreatitis [12, 40]. Koutroumpakis et al. [15] indicated that a rise in BUN at 24 h outperforms other laboratory markers in predicting persistent organ failure and pancreatic necrosis in acute pancreatitis. Our results showed a positive association between an initial increased BUN level at admission and the development of SAP in acute pancreatitis.

Pleural effusion is often observed during acute pancreatitis. A possible explanation is that pancreatic duct disruption results in leakage of pancreatic secretions directly into the peritoneal cavity via the trans-diaphragmatic lymphatic channels. Maringhini et al. [41] found that the presence of pleural effusion was associated with an increased incidence of pancreatic pseudocyst in acute pancreatitis. Heller et al. [42] demonstrated a correlation between pleural effusion on chest radiograph and severity in accordance with the Atlanta criteria. The present study suggests that pleural effusion with an OR of 4.68 (95% CI 2.42–9.05) was a strong individual predictor of SAP defined by the up-to-date revised Atlanta criteria.

The application of the proposed SABP score is expected to change current clinical practice in the management of acute pancreatitis. Patients with high SABP scores may have much more pronounced risk factors, such as SIRS, pleural effusion, elevated BUN levels and low albumin, enhancing the risk of developing SAP (Figs. 3, 4). Therefore, in order to prevent occurrence of SAP, patients with high SABP scores should be monitored more carefully or even transferred to intensive care units (for example, for respiratory support for SIRS). These patients should also receive more active intravenous fluid therapy to correct intravascular volume depletion so as to decrease high BUN levels [19]. Additional interventions could be evaluated for relevance in this setting, and especially for high SABP scores. For example, it is well established that albumin infusion improves outcome of patients with septic shock [43], liver cirrhosis with hepatorenal syndrome [44] or spontaneous bacterial peritonitis [45]. Therefore, an interesting hypothesis would be whether the administration of albumin in patients with acute pancreatitis and hypoalbuminemia could decrease mortality or prevent development of SAP, since severe acute pancreatitis shares many features with sepsis syndrome and septic shock [46]. A future study could aim to evaluate the role of albumin replacement in the treatment of acute pancreatitis with hypoalbuminemia.

There is no consensus as to the best prognostic markers in acute pancreatitis in the literature. The APACHE II score requires the collection of a large number of parameters, which makes it clinically cumbersome so that APACHE II is seldom used in clinical practice [47, 48]. The HAPS was primarily developed for rapid initial identification of patients with a first attack of acute pancreatitis who do not require intensive care but not for prediction of SAP [14]. C-reactive protein has the advantages of low cost and simple assay. Nevertheless, American College of Gastroenterology guidelines state that the utilization of C-reactive protein to predict severity in patients in AP is not practical as it takes 72 h to become accurate [7]. Mounzer et al. [3] suggested that the best classifiers in predicting the development of persistent organ failure at admission and 48 h after admission were modified Glasgow and JSS score, respectively. However, the AUC of modified Glasgow and JSS score was inferior to SABP and BISAP score in our study (Figs. 1, 5). This difference may be partly explained by the fact that we ruled out patients that had already developed organ failure at data collection, e.g. patients with PaO_2_ < 60 mmHg (respiratory failure). Which may result in a decrease of the total calculated score since PaO_2_ < 60 mmHg (respiratory failure) is one of the items included in both the Glasgow and JSS score [3]. The other possible explanation may be that these scoring systems were used as continuous variables in our study while they were converted into binary values in the study by Mounzer et al. [3] when calculating AUC. Dichotomization of a continuous predictor has many disadvantages, such as loss of information, reduction in power and increase in the probability of false positive results [30, 49].

The novelties and strengths of our study include the following: (i) To the best of our knowledge, this is the first study attempting to develop an index score using SAP defined by the up-to-date revised Atlanta criteria as the primary outcome. In addition, this is the first study to evaluate SIRS and pleural effusion as potential predictors of SAP defined by the up-to-date revised Atlanta criteria; (ii) Patients with acute pancreatitis could be divided into three groups according to different SABP scores according to CART analysis (Fig. 3), which is easy to use for risk stratification of acute pancreatitis at the bedside; (iii) SABP uses findings of vital signs, routine laboratory data, and imaging to derive a four-point score, which make it of similar simplicity to BISAP yet maintains a higher diagnostic accuracy [12]. The calculation of the modified Glasgow (eight points) and JSS score (nine points) is more complicated and these scores contain data not routinely collected at the time of hospitalization (e.g. lactate dehydrogenase, base excess, etc.); (iv)The SABP has another advantage over the Glasgow score in that it is calculated within 24 h of admission. American Gastroenterological Association Institute Guidelines propose that initial management decisions in AP can alter the course of disease and duration of hospitalization [50]. One of the expert’s opinions is that the first 24 h (“golden hours”) of care of patients with AP is crucial to reducing the morbidity and mortality. The modified Glasgow score require 48 h to complete, missing a potentially valuable early therapeutic window [12]; (v) The last advantage over other scoring systems is that SABP score could be used at different onset-to-admission times. AP is a dynamic and evolving process that involves multiple systems and the risk for organ complications [51]. We computed predicted probability of SAP over the SABP score from 0 to 10 for different onset-to-admission time (Fig. 4).

However, our study also has several limitations: Firstly, there were missing data for APACHE II, Glasgow score, JSS score and CRP in the derivation cohort due to retrospective study design, which may produce selection bias. However, the AUCs of these scores or markers were still lower than our SABP score when analysed in prospectively collected validation cohort with completed data. Secondly, we did not evaluate other putative risk factors, such as abdominal pressure and serum calcium despite the large number of candidate predictors which were examined. Lastly, radiologic scoring systems (such as computed tomography severity index, extrapancreatic score) have not been compared though many scoring systems were evaluated in our study. It will be interesting to compare our SABP score with such radiologic scoring systems in the future.

## Conclusions

In conclusion, SIRS and pleural effusion are useful predictors of SAP defined by the up-to-date revised Atlanta criteria. SABP score might be a useful tool to stratify patients at risk of developing SAP defined by the up-to-date revised Atlanta criteria and the application of it on admission may improve clinical care and management strategies in acute pancreatitis.

## Additional file

**Additional file 1: Table S1.** Univariable analysis of predictive factors of severe acute pancreatitis in derivation.

## Data Availability

The datasets used and/or analysed during the current study are available from the corresponding author on reasonable request.

## Abbreviations

ALT: alanine aminotransferase
AP: acute pancreatitis
AST: aspartate aminotransferase
AUC: area under the curve of the receiver operating characteristic curve
BISAP: bedside index of severity in acute pancreatitis
BMI: body mass index
BUN: blood urea nitrogen
CI: confidence interval
CT: computed tomography
ERCP: endoscopic retrograde cholangiopancreatography
HAPS: harmless acute pancreatitis score
IQR: interquartile range
OR: odds ratio
POF: persistent organ failure
PT: prothrombin time
ROC curve: receiver operating characteristic curve
SABP: systemic inflammatory response syndrome, albumin, blood urea nitrogen and pleural effusion
SAP: severe acute pancreatitis
SIRS: systemic inflammatory response syndrome
SD: standard deviation
