# Supplementary material for: Development and validation of a risk prediction score for severe acute pancreatitis
Source: J Transl Med. 2019 May 8;17:146. doi: 10.1186/s12967-019-1903-6 (PMC6505180; doi:10.1186/s12967-019-1903-6)
Supplement: Supplementary file 1 — Additional file 1: Table S1. Univariable analysis of predictive factors of severe acute pancreatitis in derivation. [file 12967_2019_1903_MOESM1_ESM.docx]

**Table S1.** Univariable analysis of predictive factors of severe acute pancreatitis in derivation in derivation**.**

| Variable | No-SAP (n=632) | SAP (n=68) | P values |
| --- | --- | --- | --- |
| Age ,years(IQR) | 47(37-62) | 52(39-66) | 0.15 |
| Male sex, N (%) | 395(62.5) | 40(58.8) | 0.55 |
| Duration of symptoms, days | 1.82±0.79 | 1.88±0.88 | 0.54 |
| BMI, kg/m^2^ (IQR) | 23.5(21.1-26.1) | 24.1(22.0-26.6) | 0.098 |
| SIRS, N (%) | 224(35.4) | 48(70.6) | <0.001 |
| Alcohol etiology, N (%) | 92(14.6) | 4(5.9) | 0.048 |
| Laboratory findings |  |  |  |
| Hematocrit | 0.42(0.38-0.45) | 0.44(0.40-0.47) | 0.006 |
| Platelets (10^9^/L) | 198(162-233) | 173 (134-218) | 0.007 |
| Prothrombin time, seconds(IQR) | 13.8(13.1-14.5) | 14.6(13.3-15.9) | <0.001 |
| Albumin, g/L(IQR) | 36.8(33.5-39.9) | 30.4(27.4-34.5) | <0.001 |
| Bilirubin, mg/dL(IQR) | 1.17 (0.76-1.81) | 1.14 (0.88-1.73) | 0.96 |
| ALT, U/L(IQR) | 39(18-108) | 49(26-77) | 0.36 |
| AST, U/L(IQR) | 33(20-83) | 60(38-89) | 0.0001 |
| Glucose, mg/dL(IQR) | 141(115-186) | 184(143-264) | <0.001 |
| BUN, mg/dL(IQR) | 13.2(10.4-16.5) | 20.9(14.7-33.1) | <0.001 |
| Pleural effusion, N (%) | 91(14.4) | 44(64.7) | <0.001 |

(a) Data were mean ± standard deviation, or numbers and percentages, or median (25th–75th percentile), as appropriate. (b) N, number; IQR, interquartile range; BMI, body mass index; SIRS, Systemic Inflammatory Response Syndrome; ALT, alanine aminotransferase; AST, aspartate aminotransferase; BUN, blood urea nitrogen.
